# Clinical efficacy and safety of a single administration of fluralaner injectable suspension (BRAVECTO^®^ injectable) vs. monthly administration of oral afoxolaner (NexGard^®^) in dogs for tick and flea control over one year under European field conditions

**DOI:** 10.1186/s13071-024-06590-1

**Published:** 2024-12-09

**Authors:** Ivo Petersen, Susanne Goebel-Lauth, Thierry Pobel, Maria J. Gil, Wolfgang Löhlein, Oliver Wolf, Eva Zschiesche, Bjoern Welzel, Lea Heinau

**Affiliations:** 1 MSD Animal Health Innovation GmbH, Schwabenheim, Germany; 2 TPC Biomed Veterinary Clinical Development and Biostatistics, Soria, Spain; 3 Löhlein & Wolf Vet Research and Consulting, Munich, Germany

**Keywords:** Afoxolaner, Bravecto^®^ injectable, Dog, Flea, Ticks, Fluralaner, NexGard^®^, Field study

## Abstract

**Background:**

Year-round control of canine flea and tick infestations requires owner compliance with recommendations for regular treatments. Compliance failures can result in increased exposure of dogs to tick-borne pathogens and resurgence of flea populations. This study investigated the year-long efficacy of fluralaner 150 mg/ml injectable suspension (BRAVECTO^®^ injectable), developed to remove the need for multiple owner-administered, within-year treatments.

**Methods:**

This randomized, examiner-masked, non-inferiority study enrolled household dogs at veterinary clinics in Germany, France, and Spain. Each household contained a primary dog infested with ≥ 4 ticks or ≥ 5 fleas. Additional dogs in each household received the same treatment as the primary dog, either a single injection with fluralaner (15 mg/kg) on day 0, or 12 monthly treatments with oral afoxolaner (NexGard^®^) beginning on day 0. Owners presented their dogs for tick and flea assessments at visits 2 through 10 (days 14, 28, 56, 84, 112, 224, 280, 336, 365). Primary endpoints were the percentages of primary dogs free of live ticks or fleas at visit 10. Secondary endpoints were the percentage reductions of live ticks and fleas in primary dogs. All treated dogs were observed for adverse reactions throughout the study.

**Results:**

The analyzed per-protocol population included 415 primary dogs (fluralaner 279, afoxolaner 136) from 976 treated dogs (fluralaner 653, afoxolaner 323). From visits 2 through 10, ≥ 95% of primary dogs in each group were tick-free, and ≥ 93% were flea-free. The percentage of dogs free of ticks or fleas was non-inferior (*P* ≤ 0.0048) in the fluralaner group compared to the afoxolaner group at visit 10 and all earlier visits. Compared to baseline, fluralaner-group tick and flea counts were reduced by > 99%; afoxolaner-group tick and flea counts by > 98% and > 97%, respectively. There were no unexpected adverse events in any treated dog in either group, nor any sign of interactions between concomitantly administered vaccines and medications.

**Conclusions:**

A single subcutaneous fluralaner injection provided a level of tick and flea control equivalent to that of 12 monthly administrations of afoxolaner. The sustained fluralaner efficacy helps maintain canine health by retaining treatment with the veterinarian and eliminating treatment-compliance failures by pet owners between veterinary visits.

**Graphical Abstract:**

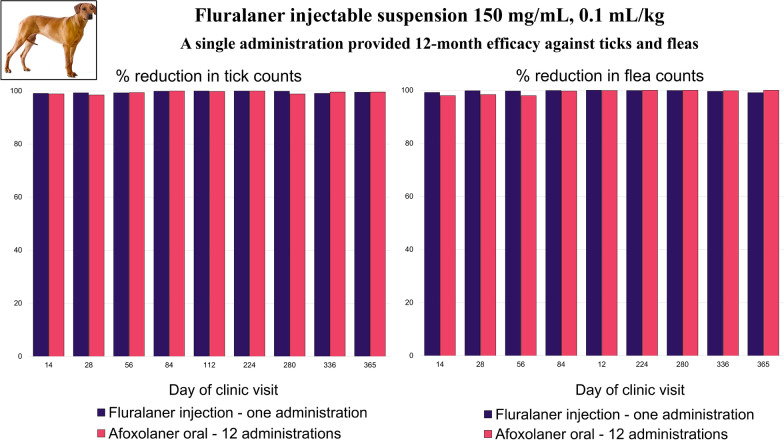

**Supplementary Information:**

The online version contains supplementary material available at 10.1186/s13071-024-06590-1.

## Background

Changing climatic conditions and anthropogenic influences have altered tick distribution, abundance, and seasonal activity over the last few decades [1]. Throughout Europe, year-round exposure to ticks and fleas, and the pathogens they carry, presents a risk to dogs and humans. The two most commonly reported tick species in Europe, each with an expanding geographical range, are *Ixodes ricinus* and *Dermacentor reticulatus* [2–5]. The former is a vector of *Borrelia* spp., *Babesia* spp., *Anaplasma phagocytophilum*, and flaviviruses that cause European tick-borne encephalitis [6–8]. *Dermacentor reticulatus*, a vector of *Babesia* spp., *Rickettsia* spp., and flaviviruses, is also seen as an increasing threat [3, 9, 10]. Other tick species of potential importance include *Rhipicephalus sanguineus*, a vector of *Babesia* spp., *Bartonella* spp., *Hepatozoon canis*, and *Ehrlichia canis*, and *Dermacentor marginalis*, while the migration of infested birds can act as a seed for establishment of new tick species [11, 12]. Those migrations, along with climate change, including warmer winters, changes to wildlife patterns, deforestation, and movement of pets with their owners are factors contributing to the geographical expansion and increased intensity of tick infestations [2, 3, 12–15]. Findings of winter activity of *I. ricinus* in Sweden and winter questing of *I. ricinus* and *D. reticulatus* in Germany provide reinforcement of recommendations for year-round tick control for dogs at risk of exposure [14–16]. *Rhipicephalus sanguineus* is an endophilic tick, i.e., often found indoors, and when dogs are constantly available as hosts, tick populations can rapidly increase, and infestations in kennels or homes are very difficult to control [17].

The cat flea, *Ctenocephalides felis*, is a vector of *Bartonella*, *Mycoplasma*, and *Rickettsia* spp. and also acts as an intermediate host of the tapeworm *Dipylidium caninum* and the filarid *Acanthocheilonema* spp. [18–20]. Flea allergy dermatitis (FAD), due to a sensitivity reaction to proteins in flea saliva, is one of the most common dermatological conditions presented to veterinarians [21, 22]. A female* C. felis* can begin egg-laying within 24–48 h after host acquisition, producing up to 50 eggs/days—eggs that then fall from a host into the environment [23]. Flea hosts include other dogs, cats, and wildlife such as foxes and rodents. Indoor environments provide an appropriate temperature and humidity for egg hatching and development, and the greatest proportion (estimated at 95%) of a flea infestation can be present as pre-adult life cycle stages in a dog’s environment [24]. Therefore, year-round flea control measures are advised for dogs at continual risk of reinfestation, including in winter months [16].

When administered according to label recommendations, the isoxazoline ectoparasiticides afoxolaner, fluralaner, sarolaner, and lotilaner have been shown to provide a high level of control of ticks and fleas in dogs [25–28]. Those recommendations are for owners to administer afoxolaner, sarolaner, and lotilaner at monthly intervals, and fluralaner at intervals of 8–12 weeks. A longer duration of action means that fewer treatments will be required to maintain protection against fleas and ticks, and fewer treatments can facilitate improved compliance with optimal ectoparasiticidal control measures [29, 30].

To further improve compliance, and to give veterinarians better control over tick and flea protection programs, an injectable formulation of fluralaner (150 mg/ml) was developed to provide a full year of efficacy. Laboratory studies showed that this formulation provides 365 days of protection against infestations with ticks (*R. sanguineus*, *I. ricinus*, *I. hexagonus*, *D. reticulatus*) and fleas [31, 32]. A field study was initiated in Europe to investigate the product’s performance under conditions of natural infestations. The objective of this study was to evaluate the 1-year efficacy against flea and tick infestations, and the safety of this fluralaner injectable suspension, administered once to client-owned dogs, compared to 12 monthly doses of afoxolaner.

### Methods

This positive-controlled, randomized, examiner-masked, multi-center study was conducted in accordance with the European Medicines Agency (EMA) and the World Association for the Advancement of Veterinary Parasitology (WAAVP) guidelines for efficacy evaluation of parasiticides against flea and tick infestation on dogs and cats [33, 34]. The study was also conducted in accordance with statistical principles for veterinary clinical trials and with good clinical practices, although in France, COVID-19 restrictions on monitoring visits placed a limitation on full alignment with the latter [34–36].

### Animals

For study enrollment, dogs presented to participating practices or clinics, including all household dogs, had to be ≥ 6 months old and weigh ≥ 2 kg, have a hair coat and temperament that would allow flea and tick count procedures, and at least one household dog had to have one or both of a live tick count ≥ 4 and a live flea count ≥ 5. A household was excluded if it contained any of the following: a severely ill dog in need of intensive veterinary care; a dog with known epilepsy; a dog that received ectoparasiticide treatment during the exclusion period; a pregnant or lactating female; more than five dogs; a history of environmental insecticidal or insect growth regulator treatments within 2 months before study start; and any non-canine pets that could harbor fleas or ticks (e.g., cats, rabbits, guinea pigs). Enrolled dogs were required to be healthy, although dogs with stable chronic medical conditions could be included at investigator discretion. Dogs with dermatological lesions believed to be linked to FAD could be included. Dogs remained in the household of their owners and were maintained in their usual environmental conditions. Grooming and bathing with non-ectoparasiticidal shampoos were allowed during the study. From enrollment until day 365 (± 3 days), owners were not allowed to administer any additional ectoparasiticidal treatments to their dogs, or to perform any premise treatments for environmental flea control. Dogs were fed, with access to water, in accordance with their normal routine. For households with multiple qualifying dogs, the investigator selected one to be the primary dog (usually the first dog in the household that qualified, based on acceptable tick or flea count).

### Treatment

Each household was randomized using computer-generated randomization lists to either a fluralaner group or an afoxolaner group in a 2:1 ratio. All dogs in a household received the same treatment, administered according to the same schedule as the primary dog. Treatments were either a single subcutaneous administration of BRAVECTO^®^ injectable, administered at a dose rate of 15 mg fluralaner kg body weight (0.1 ml/kg), or 12 monthly oral treatments with afoxolaner. The fluralaner injection was supplied as a vehicle vial and a fluralaner powder vial, which was constituted to an injectable suspension prior to administration by qualified veterinary staff. The control product, afoxolaner (NexGard^®^, chewable tablets for dogs, Merial, Lyon, France), was supplied in commercial packaging and was administered according to label directions at 28-day intervals (± 3 days), during a veterinary visit, either by veterinary clinic staff or by the owner. At each visit, each afoxolaner-group dog was observed for at least 10 min after administration for any tablet regurgitation or vomiting. Dogs in the fluralaner group were observed for any immediate reaction to injection.

### Tick and flea assessments

Study dogs were naturally infested with ectoparasites. Counts were performed according to the WAAVP guidelines, pushing the hair against its natural nap to expose the skin and any ticks, attached or unattached, or fleas [34]. All ticks were removed gently using forceps, counted, and classified as live or dead. The counting procedure began with a visual examination, starting at the head of the dog and covering all dorsal and lateral areas, after which the dog was turned gently onto its back for ventral examination. Each dog was examined in this manner for at least 5 min, after which it was combed (approximately 11–13 teeth/cm) for an additional minimum of 5 min to recover any small ticks and live fleas that may have been missed during the visual observation. The combing procedure consisted of overlapping strokes from the front (head, ears, neck, etc.) to the back including the tail, the lateral sides including the legs, chest, and the ventral sides (armpits, belly, and inner side of legs). Special attention was paid to predilection sites (hair whorls beneath the ears, the armpits and belly, the hair whorls at the caudal legs, base of the tail, and dorsum just cranial to the tail). Thus, the total examination time was at least 10 min (i.e., ≥ 5 min examination plus ≥ 5 min combing). Any ticks found by the owner between the scheduled visits and brought to the clinic were counted, and species determination was performed at a commercial laboratory (Vet Med Labor GmbH, Division of IDEXX Laboratories, Ludwigsburg, Germany). For all primary dogs presenting with marked pruritus or skin lesions considered at study inclusion to be FAD-related (erythema, papules, crusts, scales, alopecia, excoriation), a descriptive evaluation of the skin and hair examination was recorded and then, at subsequent visits, monitored for change.

Owners were required to present their primary dog for tick and flea assessments for visit 2 (day 14 ± 2) and for visits 3 through 10 (on approximately days 28, 56, 84, 112, 224, 280, 336, and 365). Tick and flea counts were not repeated for non-primary household dogs after visit 1. Afoxolaner was dispensed to owners for administration to all household dogs in that study group when treatment was scheduled for non-visit days. All other household dogs were presented at visits 3, 4, 7, and 10, or any time if an owner suspected an adverse event.

### Statistics

For efficacy assessment, an enrollment target of approximately 120 primary dogs in the fluralaner group and 60 in the afoxolaner group was set to demonstrate non-inferiority of fluralaner. This was based on an estimated efficacy (“parasite free animals”) of 95% for both fluralaner and afoxolaner, with a tolerated non-inferiority of δ = 0.10, when the (one-sided) level of significance was set to α = 0.025, with a power of 1 − β = 0.8. With an expected co-infestation rate of approximately 25% (25% of the primary dogs infested with ticks also presented with a flea infestation), 315 primary dogs were to be included (210 in the fluralaner group, 105 in the afoxolaner group), i.e., 135 primary dogs with ticks only, 135 primary dogs with fleas only, and 45 primary dogs with ticks and fleas. Assuming a drop-out rate of 25%, the targeted total enrollment was 420 primary dogs (i.e., 420 households). The experimental statistical unit was the individual animal.

For the assessment of safety and adverse events, all dogs (primary plus non-primary dogs in each household) that received at least one treatment were included. For efficacy assessments, the per-protocol population (PPP) comprised all primary dogs that were treated and examined according to the protocol. Homogeneity of study groups at inclusion was evaluated descriptively for all dogs before treatment on day 0. Frequency tables were used to compare the distribution of sex, breed, living conditions, and presence of skin lesions possibly related to FAD in both study groups.

The primary endpoints were the percentages of primary dogs free of live ticks or free of live fleas (parasite-free cases) at visit 10 (day 365). The Farrington–Manning test of non-inferiority for the risk difference was used to compare the percentage of parasite-free cases in the fluralaner and afoxolaner groups [37]. Both *P*-value and lower 97.5% one-sided confidence limits were calculated. If the lower confidence limit was above −0.10, non-inferiority was concluded. This comparison was also completed for tick and flea count time points visit 2 to visit 9. Secondary endpoints were the percentage reduction of ticks and fleas in initially infested primary dogs (day 0 tick count ≥ 4, flea count ≥ 5) from the PPP.

For secondary endpoint assessments, arithmetic and geometric means of the PPP were determined for each study group at each visit (pre-treatment at visit 1 [V1], and post-treatment at V2 to V10) and percentage reduction was calculated for each post-treatment visit as follows:

$${\text{Reduction }}\left[ \% \right]\, = \,\overline{{\text{x}}}_{{{\text{pre}}}} {-}\overline{{\text{x}}}_{{{\text{post}}}} \,{\text{x}}\, \times \,{1}00_{{{\text{pre}}}}^{ - }$$

where x̅_pre_ is the mean of live ticks/live fleas pre-treatment (V1), and x̅_post_ is the mean of live ticks/live fleas post-treatment. Geometric means were calculated using the *Y* = log_e_(x + 1) transformation.

## Results

**Fig. 1:**
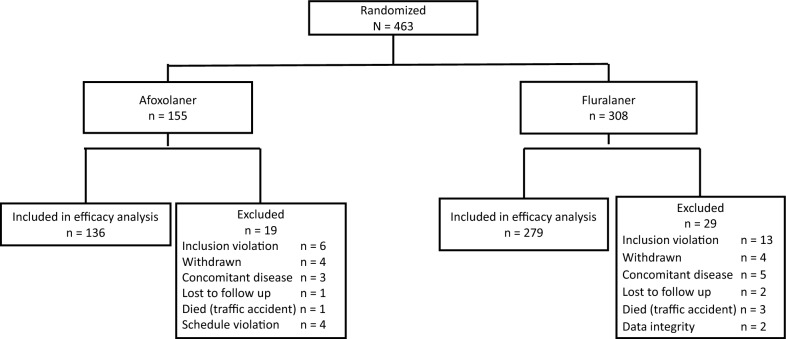
Disposition of enrolled primary dogs

Enrollments were completed during April 2021 through June, 2021, and final assessments were completed in August, 2022. From 463 households (i.e., 463 primary dogs), including additional dogs in participating households, 653 received the fluralaner injection and 323 received afoxolaner tablets. For fluralaner, mixed-breed dogs comprised 32.9% of those that were treated, and of the 116 breeds, the most often recorded were Great Anglo-French hound (56, 8.6%), Griffon Nivernais (25, 3.8%), and Labrador Retriever (22, 3.4%) (a full list of enrolled breeds is included as Additional file 1: Table S1). The smallest dog enrolled was a Chihuahua with 2.0 kg body weight, and the largest dog enrolled was a Great Dane with 74.2 kg body weight. On the day of inclusion (day 0), *I. ricinus* was identified on 309 dogs, *R. sanguineus* on 87, *D. reticulatus* on 32, and *I. hexagonus* on 15 dogs. Demographic distribution of dogs within each treatment group was well balanced in terms of dog age, sex, the presence of lesions of flea allergy dermatitis, and the number of household dogs (Table 1). With the exclusion of 48 of the 463 primary dogs from the PPP, 415 dogs were included in the statistical analysis (Fig. 1). The most common reason for exclusion was a violation of the enrollment procedure, for instance, enrollment of dogs with too few ticks or fleas, or more than five dogs per owner or per household.

**Table 1:** Demographic background of primary dogs

	Fluralaner injection	Afoxolaner oral
Number of primary dogs (households)	279	136
Mean age [years] (± SD)	5.3 (3.6)	6.1 (3.7)
Range	0.5–16	0.5–16
Mean body weight [kg] (± SD)	22.3 (12.9)	24 (12.1)
Range	2.0–66.2	2.0–66.2
Sex		
Female (% intact)	128 (26.6)	66 (39.4)
Male (% intact)	151 (35.8)	70 (48.6)
FAD-related skin lesions		
No (%)	261 (93.5)	128 (94.1)
Yes (%)	18 (6.5)	8 (5.9)
Mean number of dogs per household (± SD)	2.1 (1.4)	2.1 (1.4)
Number of household dogs		
1	144 (51.6%)	70 (51.5%)
2	50 (17.9%)	27 (19.9%)
3	29 (10.4%)	13 (9.6%)
4	22 (7.9%)	11 (8.1%)
5	34 (12.2%)	15 (11.0%)

*SD* standard deviation

Abnormal clinical observations at inclusion were made in 9.5% of the animals of the fluralaner group and in 8.9% of the afoxalaner group. Abnormalities mostly comprised skin diseases, arthrosis, arthritis, lameness, spondylosis, teeth and mouth diseases, hypothyroidism, diseases of the eyes, and cardiovascular diseases. Treatments of these findings were continued during the study, e.g., with bedinvetmab, carprofen, galliprant, or levothyroxine sodium.

On day 0, 236 dogs in the PPP met the inclusion criterion of infestation with ≥ 4 ticks (164 and 72 in the fluralaner and afoxolaner groups, respectively). By country, 76.7% of those dogs were from Germany, 14.4% from Spain, and 8.9% from France. The criterion of ≥ 5 fleas was met by 200 dogs in the PPP (fluralaner 128, afoxolaner 72), 40.0% from Spain, 34.0% from France, and 26.0% from Germany. Co-infestation criteria for fleas and ticks was met by 21 dogs in the PPP, a rate of approximately 5%, considerably lower than the expected co-infestation rate of 25%.

From visits 2 (the first post-treatment visit) through 10, the percentage of tick-free primary dogs exceeded 95% in both groups (Table 2). Similarly, the proportion of flea-free primary dogs exceeded 95% at all time points except for visit 2 in the fluralaner group (93.8% flea-free) and visits 2 and 4 in the afoxolaner group (93.1% flea-free at both visits) (Table 3). The percentages of tick-free and flea-free primary dogs were significantly non-inferior (*P* ≤ 0.0048) in the fluralaner injection group compared to the afoxolaner group for the primary efficacy endpoint (visit 10) and at visits 2 through 9.

**Table 2:** Percentage of tick-free primary dogs (dogs with ≥ 4 ticks on day 0)

Visit (study day)	Tick-free dogs		*P*-value	Lower 97.5% one-sided confidence interval
	Fluralaner injection (*n* = 164)	Afoxolaner oral (*n* = 72)		
2 (14)	158 (96.3%)	69 (95.8%)	< 0.0001	−0.0514
3 (28)	161 (98.2%)	69 (95.8%)	< 0.0001	−0.0319
4 (56)	158 (96.9%)^a^	69 (95.8%)	< 0.0001	−0.0452
5 (84)	163 (99.4%)	72 (100.0%)	< 0.0001	−0.0520
6 (112)	164 (100.0%)	71 (98.6%)	< 0.0001	−0.0350
7 (224)	164 (100.0%)	72 (100.0%)	Statistical test not possible	
8 (280)	163 (99.4%)	70 (97.2%)	< 0.0001	−0.0302
9 (336)	157 (95.7%)	71 (98.6%)	0.0026	−0.0788
10 (365)	160 (97.6%)	70 (97.2%)	< 0.0001	−0.0493

^a^ Assessment from 163 dogs

**Table 3:** Percentage of flea-free primary dogs (dogs with ≥ 5 fleas on day 0)

Visit (study day)	Flea-free dogs		*P*-value	Lower 97.5% one-sided confidence interval
	Fluralaner injection (*n* = 128)	Afoxolaner oral (*n* = 72)		
V2 (14)	120 (93.8%)	67 (93.1%)	0.0019	−0.0653
V3 (28)	125 (97.7%)	70 (97.2%)	0.0003	−0.0551
V4 (56)	124 (96.9%)	67 (93.1%)	< 0.0001	−0.0310
V5 (84)	127 (99.2%)	69 (95.8%)	< 0.0001	−0.0279
V6 (112)	127 (100.0%)^a^	71 (98.6%)	< 0.0001	−0.0416
V7 (224)	127 (99.2%)	71 (98.6%)	< 0.0001	−0.0494
V8 (280)	127 (99.2%)	72 (100.0%)	0.0003	−0.0598
V9 (336)	81 (95.3%)	70 (97.2%)	0.0045	−0.0798
V10 (365)	124 (96.9%)	72 (100.0%)	0.0048	−0.0832

^a^Assessment from 127 dogs

Secondary efficacy endpoints were based on the percentage reduction of live ticks and fleas in initially infested primary dogs. At all post-treatment time points in the fluralaner group, the arithmetic and geometric mean reductions in tick and flea counts were reduced by > 99% (Tables 4 and 5). At all post-treatment time points in the afoxolaner group, the tick and flea count reductions were > 98% and > 97%, respectively (both > 99% by geometric means).

**Table 4:** Mean tick counts and percentage reduction from day 0 in live tick counts at each visit

Visit (day)	1 (0)	2 (14)	3 (28)	4 (56)	5 (84)	6 (112)	7 (224)	8 (280)	9 (336)	10 (365)
Fluralaner										
AM (GM)	6.7 (6.0)	0.1 (0.0)	0.1 (0.0)	0.0 (0.0)	0.0 (0.0)	0.0 (0.0)	0.0 (0.0)	0.0 (0.0)	0.1 (0.0)	0.0 (0.0)
SD	4.5	0.4	0.4	0.3	0.1	0.0	0.0	0.1	0.3	0.2
Median	5.0	0.0	0.0	0.0	0.0	0.0	0.0	0.0	0.0	0.0
Range	4–36	0–4	0–4	0–3	0–1	0–0	0–0	0–1	0–3	0–2
% efficacy AM (GM)		99.1 (99.4)	99.3 (99.6)	99.3 (99.5)	99.9 (99.9)	100.0 (100.0)	100.0 (100.0)	99.9 (100.0)	99.1 (99.4)	99.5 (99.7)
Afoxolaner										
AM (GM)	6.5 (5.9)	0.1 (0.0)	0.1 (0.1)	0.0 (0.0)	0.0 (0.0)	0.0 (0.0)	0.0 (0.0)	0.1 (0.0)	0.0 (0.0)	0.0 (0.0)
SD	4.4	0.4	0.5	0.2	0.0	0.1	0.0	0.5	0.2	0.2
Median	6.0	0.0	0.0	0.0	0.0	0.0	0.0	0.0	0.0	0.2
Range	4–39	0–3	0–4	0–1	0–0	0–1	0–0	0–4	0–2	0–1
% efficacy AM (GM)		98.9 (99.3)	98.5 (99.1)	99.4 (99.5)	100.0 (100.0)	99.8 (99.8)	100.0 (100.0)	98.9 (99.5)	99.6 (99.7)	99.6 (99.7)

**Table 5:** Mean flea counts and percentage reduction from day 0 in live flea counts at each visit

Visit (day)	1 (0)	2 (14)	3 (28)	4 (56)	5 (84)	6 (112)	7 (224)	8 (280)	9 (336)	10 (365)
Fluralaner										
AM (GM)	13.9 (11.4)	0.1 (0.1)	0.0 (0.0)	0.0 (0.0)	0.0 (0.0)	0.0 (0.0)	0.0 (0.0)	0.0 (0.0)	0.1 (0.0)	0.1 (0.0)
SD	12.9	0.5	0.2	0.2	0.1	0.0	0.2	0.1	0.3	0.8
Median	10.0	0.0	0.0	0.0	0.0	0.0	0.0	0.0	0.0	0.0
Range	5–110	0–4	0–1	0–2	0–1	0–0	0–2	0–1	0–2	0–6
% efficacy AM (GM)		99.2 (99.4)	99.8 (99.9)	99.7 (99.8)	99.9 (100.0)	100.0 (100.0)	99.9 (99.9)	99.9 (100.0)	99.6 (99.7)	99.1 (99.6)
Afoxolaner										
AM (GM)	14.3 (5.9)	0.3 (0.0)	0.2 (0.1)	0.3 (0.0)	0.0 (0.0)	0.0 (0.0)	0.0 (0.0)	0.0 (0.0)	0.0 (0.0)	0.0 (0.0)
SD	20.1	1.5	1.9	1.8	0.2	0.1	0.1	0.0	0.2	0.0
Median	8.0	0.0	0.0	0.0	0.2	0.0	0.0	0.0	0.0	0.0
Range	5–143	0–12	0–16	0–15	0–1	0–1	0–1	0–0	0–1	0–0
% efficacy AM (GM)		98.0 (99.0)	98.4 (99.5)	98.0 (99.1)	99.7 (99.7)	99.9 (99.9)	100.0 (99.9)	100.0 (100.0)	99.8 (99.8)	100.0 (100.0)

*AM* arithmetic mean, *GM* geometric mean, *SD* standard deviation

At the inclusion visit, 33 primary dogs presented with skin lesions (erythema, crusts, scales) with a possible relation to FAD (fluralaner, 23 dogs; afoxolaner, 10 dogs). In the fluralaner-treated dogs, at visit 2 (day 14), those skin lesions had resolved to normal in 10 dogs (43.5%), at visit 3 (day 28) in an additional eight dogs (34.8%), at visit 4 (day 56) in three dogs (13.0%), and at visit 5 (day 84) in one dog (4.3%). On owner request, one dog was removed at visit 4 without improvement of the FAD-related lesions. In the afoxolaner-treated primary dogs, FAD-related skin lesions resolved to normal at visit 2 in five dogs (50.0%), at visit 3 in two dogs (20.0%), at visit 4 in one dog (10.0%), and at visit 5 in two dogs (20.0%).

### Safety and concomitant medications

From the overall enrollment of 976 dogs (primary dogs plus additional household dogs), 580 adverse events were reported, irrespective of causality, over the course of the 365-day study. In the fluralaner group, 365 events occurred in 198 dogs (30.3% of fluralaner-treated dogs). In the afoxolaner group, there was a slightly higher percentage of dogs with adverse events (35.3%), with 215 events occurring in 114 treated dogs. The most frequently recorded events were those involving the gastrointestinal tract (vomiting, diarrhea), decreased appetite, skin problems (pruritus, otitis externa), and tiredness. Only tiredness in four dogs and decreased appetite in two dogs were considered related to fluralaner treatment. Five reports were received of transient self-limited pain at the fluralaner injection site related to the needle penetration of the skin during injection. No injection site swellings, erythema, or heat were observed. Suspected signs of neurological impairment were observed by the owners of single dogs from both groups. While those signs align with a warning on the product labels of isoxazoline products, none of the study events were considered treatment-related [38–40].

### Concomitant medications

Vaccines were administered to 121 fluralaner-treated dogs, and 231 different pharmaceutical (and medicinal) products and 140 different drugs or drug combinations were administered to 166 fluralaner-treated dogs. The vaccines administered to fluralaner-treated dogs were to protect against rabies, distemper, adenovirus, parvovirus, parainfluenza type 2, leptospirosis, *Borrelia* spp., *Bordetella*, and *Leishmania*. The active medicinal ingredients most frequently administered to fluralaner-treated dogs were as follows: amoxicillin alone or in combination with clavulanic acid (*n* = 86); the combination of milbemycin oxime and praziquantel (*n* = 61); meloxicam (*n* = 60); carprofen (*n* = 24); the combination of praziquantel, pyrantel, and febantel (*n* = 19); propofol (*n* = 18); doxycycline (*n* = 18); maropitant (*n* = 17); cefalexin (*n* = 15); bedinvetmab (*n* = 14) and butorphanol (*n* = 14) (see Additional file 2: Table S2 for a full listing of concomitant medications). There was no evidence of an interaction between fluralaner treatment and vaccination in any of the 121 vaccinated dogs, nor was there any evidence to indicate incomplete vaccine protection or the occurrence of a disease against which a vaccination was given. Furthermore, there was no evidence of an interaction between fluralaner treatment and the administration of concomitant medications used in the study.

## Discussion

Infestations of at least four and up to 39 ticks at the time of each dog’s enrollment show that the study was undertaken in tick-endemic areas, over at least two seasons when conditions would favor tick development. Under those challenging conditions, a single treatment with the fluralaner injection, or 12 monthly oral treatments with afoxolaner, kept ≥ 95% of dogs free of ticks for a full year and provided tick-count reductions of ≥ 99% for fluralaner and ≥ 98% for afoxolaner.

Flea counts of dogs in the PPP ranged on day 0 from at least 5 to 110 and 143 in the fluralaner and afoxolaner groups, respectively. In the dogs’ home environments, those fleas would have already been producing eggs, resulting in a continuous infestation challenge as they hatched and developed. Under those conditions, both products maintained ≥ 93% of dogs free of fleas for the full year, with flea count reductions of ≥ 99% for fluralaner and ≥ 98% for afoxolaner. The resolution of the signs of FAD in all but one dog in the fluralaner group is consistent with other studies showing the benefit of eliminating flea infestations [41–44].

A program of repeated monthly administrations of pulicides to dogs has been shown to result in a steady depletion, leading to extinction, of in-home flea challenges [41–44]. The success of such programs depends upon owner adherence to those monthly treatments, as a delayed or missed dose can result in a resurgence of a dwelling’s flea population [20]. By providing a full year’s protection, and administering treatment under veterinary supervision, fluralaner injectable suspension removes the risk of owner compliance failure. For ticks, an owner’s failure to re-treat at the required intervals can increase the risk that a dog will be exposed to tick-borne pathogens.

An important consideration for any novel drug product recently introduced into the marketplace concerns safety. The fluralaner injection was administered to 116 breeds of dogs, ranging in age from 0.5 to 16 years. All excipients in the formulation are used in a variety of previously approved injectable medications. There was no evidence of any breed or age susceptibility to adverse events. Based on individual case assessments of all reported adverse events, including the temporal relationship to treatment, only tiredness in four of 653 fluralaner-treated dogs (0.6%) and decreased appetite in two dogs (0.3%) were considered related to the fluralaner injectable treatment, and all these clinical signs resolved without any treatment in a very short time frame. There was no clinical evidence of any adverse reactions to the subcutaneous injection of fluralaner. Furthermore, there were no signs of negative interactions between the fluralaner injection and any of the commonly administered canine vaccines, nor of a negative interaction between the fluralaner injection and concomitant medications administered during the study.

This is the first study to investigate the use of this novel formulation under field conditions. The results align with laboratory studies showing that a single treatment of dogs with the fluralaner injection provided a full year of protection against repeated challenge with ticks (the brown dog tick, *R. sanguineus*) and fleas [31]. The findings of the present study show that in addition to providing 365 days of control of fleas, this fluralaner injectable suspension is also effective against *I. ricinus*, *R. sanguineus*, *D. reticulatus*, and *I. hexagonus* under field conditions.

## Conclusions

A single administration of fluralaner injectable suspension (BRAVECTO^®^ injectable) to dogs was well tolerated and resulted in ≥ 99% reduction from baseline in tick and flea counts at all time points throughout the year following treatment. At all post-treatment time points, ≥ 95% of fluralaner-treated dogs were free of ticks, and ≥ 93% were free of fleas. The single fluralaner injection treatment provided a level of tick and flea control that was at least equivalent to the control provided by 12 monthly oral administrations of afoxolaner. The sustained fluralaner efficacy can help maintain canine health by retaining treatment with the veterinarian and eliminating risk of owner treatment-compliance failures.

## Supplementary Information

Additional file 1.

Additional file 2.

## Data Availability

No datasets were generated or analyzed during the current study.

## Abbreviations

FAD: Flea allergy dermatitis
PPP: Per-protocol population
WAAVP: World Association for the Advancement of Veterinary Parasitology
