# Supplementary material for: Clinical efficacy and safety of a single administration of fluralaner injectable suspension (BRAVECTO® injectable) vs. monthly administration of oral afoxolaner (NexGard®) in dogs for tick and flea control over one year under European field conditions
Source: Parasit Vectors. 2024 Dec 9;17:504. doi: 10.1186/s13071-024-06590-1 (PMC11626764; doi:10.1186/s13071-024-06590-1)
Supplement: Supplementary file 1 — Additional file 1. [file 13071_2024_6590_MOESM1_ESM.docx]

**Additional file 1: Table S1**. Breeds (number in each) of dogs treated with fluralaner injectable

| Akita | 1 | German Spaniel | 1 |
| --- | --- | --- | --- |
| Alano Español | 1 | German Spitz | 3 |
| American Cocker Spaniel | 1 | German Wire-haired Pointing Dog | 5 |
| American Staffordshire Terrier | 1 | Giant Schnauzer | 1 |
| Anglo-French Hound | 2 | Golden Retriever | 10 |
| Australian Shepherd | 10 | Great Anglo-French Hound | 56 |
| Basque Shepherd Dog | 3 | Great Dane | 4 |
| Basset Hound | 6 | Griffon | 2 |
| Beagle | 8 | Griffon Nivernais | 25 |
| Beauceron | 2 | Griffon Vendeen | 3 |
| Belgian Shepherd Dog | 7 | Havanese | 1 |
| Bernese Mountain Dog | 2 | Hungarian Pointing Dog | 1 |
| Bloodhound | 2 | Irish Terrier | 1 |
| Blue Gascony Griffon | 2 | Italian Corso Dog | 3 |
| Blue Gascony Hound | 2 | Jack Russell Terrier | 11 |
| Bobtail | 1 | Kleiner Münsterländer | 2 |
| Border Collie | 16 | Kromfohrländer | 1 |
| Border Terrier | 1 | Labrador Retriever | 22 |
| Boxer | 2 | Large Munsterlander | 2 |
| Brittany Spaniel | 11 | Leonberger | 1 |
| Cairn Terrier | 1 | Leonese Shepherd | 15 |
| Canarian Warren Hound | 1 | Mastiff | 12 |
| Cavalier King Charles Spaniel | 1 | Medium-sized Anglo-French Hound | 1 |
| Chihuahua | 3 | Miniature Pinscher | 1 |
| Chow Chow | 1 | Norfolk Terrier | 2 |
| Collie | 3 | Petit Basset Griffon Vendeen | 1 |
| Dachshund | 11 | Pinscher | 2 |
| Dalmatian | 1 | Pointing dog | 2 |
| Deerhound | 1 | Poodle | 4 |
| Deutsch Langhaar | 2 | Porcelaine | 1 |
| Dobermann | 1 | Portuguese Warren Hound | 2 |
| Dogo Argentino | 2 | Pug | 1 |
| Dutch Schapendoes | 1 | Pyrenean Mountain Dog | 1 |
| English Cocker Spaniel | 4 | Pyrenean Sheepdog | 3 |
| English Setter | 12 | Rhodesian Ridgeback | 1 |
| Estrela Mountain Dog | 1 | Romagna Water Dog | 1 |
| Fox Terrier | 4 | Rottweiler | 1 |
| French Bulldog | 4 | Schnauzer | 1 |
| Gascon saintongeois | 13 | Sheepdog | 1 |
| German Hound | 3 | Shih Tzu | 1 |
| German Hunting Terrier | 3 | Siberian Husky | 1 |
| German Shepherd Dog | 14 | Spaniel | 1 |
| German Short-haired Pointing Dog | 7 | Spanish Greyhound | 3 |
| Shetland Sheepdog | 2 | Spanish Hound | 11 |
| Spanish Mastiff | 2 | Tibetan Terrier | 1 |
| Spanish Waterdog | 2 | Warren Hound | 13 |
| Springer Spaniel | 1 | Weimaraner | 2 |
| Staffordshire Bull Terrier | 1 | Wire-haired Pointing Griffon Korthals | 1 |
| Terrier | 1 | Yorkshire Terrier | 12 |
|  |  | Mixed breed | 215 |
