# Supplementary material for: Clinical efficacy and safety of a single administration of fluralaner injectable suspension (BRAVECTO® injectable) vs. monthly administration of oral afoxolaner (NexGard®) in dogs for tick and flea control over one year under European field conditions
Source: Parasit Vectors. 2024 Dec 9;17:504. doi: 10.1186/s13071-024-06590-1 (PMC11626764; doi:10.1186/s13071-024-06590-1)
Supplement: Supplementary file 2 — Additional file 2. [file 13071_2024_6590_MOESM2_ESM.docx]

**Additional File 2: Table S2.** Concurrent medications administered to dogs treated with the fluralaner long-acting injection during the year following treatment.

A concurrent medication was administered to 166 fluralaner-treated dogs.

| **Drug name** | **Generic name** | **Number of cases** |
| --- | --- | --- |
| Alfaxan | Alfaxolone | 5 |
| Allopurinol | Allopurinol | 1 |
| Allermyl lotion | Mono-, oligosaccharide, linolenic acid | 1 |
| Amoxiclav | Amoxicillin, clavulanic acid | 13 |
| Anesketin | Ketamine | 1 |
| Aniclindan | Clindamycin | 1 |
| Animeloxan | Meloxicam | 4 |
| Antisedan | Atipamezole | 1 |
| Apomorphine hydrochloride | Apomorphine hydrochloride | 1 |
| Apoquel | Oclacitinib | 4 |
| Atipam | Atipamezole | 2 |
| Augmentin | Amoxicillin, clavulanic acid | 1 |
| Aurimed Ear Cleaner | Panthenol, urea, lactic acid, (…) | 1 |
| Aurizon Ear Drops | Marbofloxacin, clotrimazole, dexameth. | 1 |
| Banminth Plus | Epsiprantel, pyrantel | 1 |
| Betadine Topic | Povidone iodine | 3 |
| Betafuse | Fucidin acid, betamethasone | 1 |
| Bioclamox | Amoxicillin, clavulanic acid | 2 |
| Bupredine | Buprenorphine | 2 |
| Buprenodale | Buprenorphine | 2 |
| Bupresol | Buprenorphine | 2 |
| Buscopan | Butyl scopolamine, metamizole | 1 |
| Butomidor/Butorphanol | Butorphanol | 10 |
| Butorgesic | Butorphanol | 1 |
| Butyl scopolamine | Butyl scopolamine | 2 |
| Calmin | MCT-oil, CBT 5% | 1 |
| Calmivet | Acepromazine | 1 |
| Canicur/Canikur Pro | Enterococcus faecium, bentonite | 3 |
| Canidiarix | Sulfaguanidine framycetin atropine | 1 |
| Caniprevent | Chlorothymol, isopropanol, glycerol | 3 |
| Canitenol BT | Praziquantel | 7 |
| Cardalis | Benazepril, spironolactone | 1 |
| Cardisure | Pimobendane | 1 |
| Carprotab | Carprofen | 5 |
| Carprovex | Carprofen | 3 |
| Carprox Vet | Carprofen | 11 |
| Cefalexin | Cefalexin | 1 |
| Cefazolin Normon | Cefazolin | 1 |
| Cefenidex | Chloramphenicol, dexamethasone | 1 |
| Cepetor | Medetomidine | 2 |
| Cerenia | Maropitant | 7 |
| Charcole Tablets | Charcole | 1 |
| Cimalgex Tablets | Cimicoxib | 9 |
| Clavaseptin | Amoxicillin, clavulanic acid | 3 |
| Clavobay | Amoxicillin, clavulanic acid | 3 |
| Clavudale | Amoxicillin, clavulanic acid | 2 |
| Clindabactin | Clindamycin | 1 |
| Comfortan | Methadone | 2 |
| Convenia | Cefovecin | 2 |
| Cortavance | Hydrocortisone | 4 |
| Cortizeme | Neomycin prednisolone | 3 |
| Cyclosporine Topical | Cyclosporine | 1 |
| Cylanic | Amoxicillin, clavulanic acid | 1 |
| Depo-Medrate | Methylprednisolonacetate | 1 |
| Dermamycin Skin | Neomycin, hydrocortisone, lidocaine | 3 |
| Dermipred | Prednisolone | 7 |
| Dexadreson | Dexamethason | 1 |
| Dexalon | Dexamethason | 1 |
| Diarsanyl | Electrolytes | 1 |
| Diatab | Dietary supplements | 2 |
| Diazedor | Diazepam | 1 |
| Diet Hills ZD | Hydrolyzed diet / dietary supplements | 1 |
| Domitor | Medetomidine | 5 |
| Dosalid | Epsiprantel, pyrantel | 3 |
| Douxo Calm Shampoo | Ophytrium | 2 |
| Douxo Pyo Shampoo | Chlorhexidine diglyconate | 2 |
| Douxo Calm Microemulsion | Phytosphingosine salicyloyl | 1 |
| Doxybactin | Doxycyclin | 1 |
| Doxyval | Doxycyclin | 17 |
| Duphamox | Amoxicillin | 10 |
| Efex | Marbofloxacin | 1 |
| Esaotic | Hydrocortisone, miconazole, gentamycin | 1 |
| Emedog | Apomorphine | 1 |
| Emeprid | Metoclopramide | 4 |
| Emex | Maropitant | 1 |
| Enrofloxacin | Enrofloxacin | 1 |
| Enrotab | Enrofloxacin | 2 |
| Enteritab | Enterococcus faecium, trisodium citrate | 1 |
| Enterogast | Ratanhia extract | 2 |
| Enterokur | Enterococcus faecium | 2 |
| Enterokur Pro | Enterococcus faecium | 1 |
| Entero-Teknosal | Silicon dioxide | 1 |
| Epiotic | EDTA, salicylic acid | 1 |
| Estifor | Dietary supplements | 3 |
| Euphravet | Euphrasia extract, rosae aetheroleum | 2 |
| Exitel | Praziquantel, pyrantel, febantel | 3 |
| Extronel | Praziquantel, pyrantel, febantel | 4 |
| Fentadon | Fentanyl | 1 |
| Flagyl Suspension | Metronidazole | 1 |
| Flora Complex | Probiotics | 1 |
| Floxal | Ofloxacin | 1 |
| Forthyron | Levothyroxine | 2 |
| Fradexam collyre | Framycetin dexamethason | 1 |
| Furotab | Furosemide | 1 |
| Gabapentin | Gabapentin | 2 |
| Galastop | Cabergoline | 1 |
| Galliprant | Gapiprant | 1 |
| Gentamicin-Pos | Gentamicin | 1 |
| Glucantime | N-Methylglucamin-Antimonat | 2 |
| H2-Blocker Ratiopharm | Cimetidine | 1 |
| Healing Clay | Dietary supplement | 1 |
| Hexoclean Ear Cleaner | Chlorhexidine, propylene glycol, (…) | 1 |
| Hyalutidin | Hyaluronic acid, chondroitin sulfate, (…) | 1 |
| Hydrocortisel | Neomycin, dexamethasone | 2 |
| Inflacam | Meloxicam | 2 |
| Isaderm | Fucidin acid, betamethasone | 1 |
| Isathal | Fucidin acid | 3 |
| Isoflurane | Isoflurane | 9 |
| Isopto-Max Eye Ointment | Dexamethasone, neomycin, polymyxin B | 1 |
| Kaopectate | Kaolin pectine | 1 |
| Kesium | Amoxicillin, clavulanic acid | 29 |
| Ketamine | Ketamine | 2 |
| Lanso Tad | Lansoprazole | 2 |
| Librela | Bedinvetmab | 14 |
| L-Polamivet | Levomethadone | 1 |
| Malaseb Shampoo | Miconazole, chlorhexidine | 1 |
| Manuka Lind Ointment | Manuka honey | 4 |
| Marbocyl | Marbofloxacin | 1 |
| Marbosyva | Marbofloxacin | 1 |
| Mcepe | Metoclopramide | 1 |
| Medetor | Medetomidine | 1 |
| Melosulute | Meloxicam | 8 |
| Melosus | Meloxicam | 11 |
| Melovem | Meloxicam | 2 |
| Meloxidolor | Meloxicam | 2 |
| Meloxidyl | Meloxicam | 9 |
| Meloxoral | Meloxicam | 6 |
| Metacam | Meloxicam | 21 |
| Metamizole | Metamizole | 2 |
| Metrobactin | Metronidazole | 2 |
| Metrotab | Metronidazole | 1 |
| Metrovis | Metronidazole | 2 |
| Micocep Shampoo | Miconazole, chlorhexidine | 1 |
| Milbactor | Milbemycin oxime, praziquantel | 3 |
| Milbemax | Milbemycin oxime, praziquantel | 24 |
| Milprazon | Milbemycin oxime, praziquantel | 29 |
| Milpro | Milbemycin oxime, praziquantel | 5 |
| Mitex | Miconazole, polymyxin B | 1 |
| Mopral | Omeprazole | 1 |
| Natrium chloride solution | Natrium chloride | 1 |
| Nandrosol | Nandrolone | 2 |
| Narcofol | Propofol | 5 |
| Nicilan | Amoxicillin, clavulanic acid | 4 |
| Novacen | Novminsulfone (= metamizole sodium) | 3 |
| Novalgin | Metamizole | 2 |
| Omep/Omeprazol | Omeprazole | 4 |
| Onsior | Robenacoxib | 2 |
| Ophtocycline | Chlortetracycline | 2 |
| Oralade | Electrolytes | 1 |
| Osurnia | Terbinafine, florfenicol, betamethasone | 13 |
| Ototop | Miconazole, prednisolone, polymyxin B | 1 |
| Pantoprazole | Pantoprazole | 2 |
| Peritol | Cyproheptadine | 1 |
| Phenoleptil | Phenobarbital | 1 |
| Phenpred | Prednisolone, phenylbutazone | 3 |
| Phenylbutazone | Phenylbutazone | 1 |
| Phosphaluvet | Aluminium phosphate | 3 |
| Posatex | Orbifloxacin, mometasone, posaconazole | 1 |
| Praziquantel | Praziquantel | 4 |
| Prazitel | Praziquantel, pyrantel, febantel | 7 |
| Prednisolone acetate suspension | Prednisolone | 7 |
| Prednitab | Prednisolone | 2 |
| Previcox | Firocoxib | 4 |
| Prevomax | Maropitant | 8 |
| Primazym | Pancreatic enzymes | 1 |
| Propentotab | Propentofylline | 1 |
| Propofol Lipuro | Propofol | 1 |
| Propovet | Propofol | 12 |
| Pulmofer | Dietary supplements | 1 |
| Rebohaxanid Gel | Polyhexanide | 2 |
| Recicort | Triamcinolone acetonide, salicylic acid | 1 |
| Redonyl Ultra | Palmitoylethanolamide | 1 |
| Relaxan Forte | L-Tryptophane | 2 |
| Release | Pentobarbital | 1 |
| Renes viscum | Herbal extracts | 1 |
| Respirax | Herbal extracts | 3 |
| Revertor | Atipamezole | 2 |
| Rheumocam | Meloxicam | 1 |
| Rilexine | Cefalexin | 11 |
| Rimadyl | Carprofen | 3 |
| Rimifin | Carprofen | 2 |
| Sangostyptal | Herbal extracts | 2 |
| Sedadex | Dexmedetomidine | 13 |
| Sedator | Medetomidine | 1 |
| Sivomixx | Probiotics | 1 |
| Socatyl SFD | Formosulfathizole | 1 |
| Sonotix | Ethoxydiglycol, capric glycerides, (…) | 2 |
| Spizobactin | Spiramycin, metronidazole | 1 |
| Sterofundin | Electrolytes | 3 |
| Sterovet | Electrolytes | 1 |
| Strantel | Praziquantel, pyrantel, febantel | 2 |
| Sucrabest | Sucalfate | 1 |
| Sulphix | Trimethoprim, sulphadoxine | 1 |
| Suprelorin | Deslorelin | 3 |
| Surgibond | N-butyl cyanoacrylate | 1 |
| Surolan | Prednisolone, polymyxin B, miconazole | 2 |
| Synulox | Amoxicillin, clavulanic acid | 15 |
| Tarantula Cubensis D6 | Tarantula extract | 1 |
| Terbutaline | Terbutaline | 1 |
| Tevemyxin | Neomycin, polymyxin B, acetylcysteine | 1 |
| Therios | Cefalexin | 3 |
| Tobradexa Eye Drops | Tobramycin, dexamethasone | 1 |
| Tobrex Eye Drops | Tobramycin | 2 |
| Torbugesic | Butorphanol | 3 |
| Tralieve | Tramadol | 2 |
| Traumeel | Herbal extracts | 2 |
| Triz EDTA | Tromethamine, di sodium EDTA, (…) | 1 |
| TSO | Trimethoprim, sulphadiazine | 2 |
| Urbason Injectable | Methylprednisolone | 2 |
| Ursovit ADEC | Vitamins | 1 |
| Vet Sept Ointment/Solution | Povidone iodine | 2 |
| Vetedine | Povidone iodine | 1 |
| Vetfluran | Isoflurane | 2 |
| Vetmedin | Pimobendane | 1 |
| Vetemex | Maropitant | 1 |
| Veyxyl LA | Amoxicillin | 3 |
| Viacutan Plus | Dietary supplements | 3 |
| Vitamin B / Complex | B vitamins | 2 |
| Vitamin B12 | Vitamin B12 | 1 |
| Vitamin K1 | Vitamin K1 | 3 |
| Vitofyllin | Propentofylline | 3 |
| Vomend | Metoclopramide | 1 |
| Vomisan | Dietary supplements | 1 |
| Vulnocyn Hydrogel | Active chlorine | 1 |
| Wedegest / Plus | Pancreatic enzymes | 2 |
| Wethyrox | Thyroxine | 1 |
| Ziapam | Diazepam | 2 |
| Zodon | Clindamycin | 3 |
| Zoletil | Zolazepam, tiletamine | 5 |
| Zoolac Propaste | Enterococcus spp. | 1 |
| Zykiall | Praziquantel, pyrantel, febantel | 3 |
| Name not available | Name not available | 2 |
